# Association of Acute Upper Respiratory Tract Infections with Sudden Sensorineural Hearing Loss: A Case-Crossover, Nationwide, Population-Based Cohort Study

**DOI:** 10.3390/ijerph182010745

**Published:** 2021-10-13

**Authors:** Chuan-Yi Lin, Po-Hsiu Kuo, Szu-Yuan Wu

**Affiliations:** 1 Institute of Epidemiology and Preventive Medicine, College of Public Health, National Taiwan University, Taipei 100, Taiwan; dapl0814@gmail.com; 2 Department of Public Health, College of Public Health, National Taiwan University, Taipei 100, Taiwan; 3 Department of Otorhinolaryngology, Lo-Hsu Medical Foundation, Lotung Poh-Ai Hospital, Yilan 256, Taiwan; 4 Department of Psychiatry, National Taiwan University Hospital, Taipei 100, Taiwan; 5 Department of Food Nutrition and Health Biotechnology, College of Medical and Health Science, Asia University, Taichung 413, Taiwan; 6 Big Data Center, Lo-Hsu Medical Foundation, Lotung Poh-Ai Hospital, Yilan 256, Taiwan; 7 Division of Radiation Oncology, Lo-Hsu Medical Foundation, Lotung Poh-Ai Hospital, Yilan 256, Taiwan; 8 Department of Healthcare Administration, College of Medical and Health Science, Asia University, Taichung 413, Taiwan; 9 Graduate Institute of Business Administration, Fu Jen Catholic University, Taipei 242062, Taiwan; 10 Centers for Regional Anesthesia and Pain Medicine, Wan Fang Hospital, Taipei Medical University, Taipei 110, Taiwan

**Keywords:** sudden sensorineural hearing loss, acute upper respiratory tract infections, risk, case-crossover, young age

## Abstract

The etiology of sudden sensorineural hearing loss (SSNHL) has been unclear until now. Understanding its potential etiology is crucial for the development of preventive medicine. In this study, we investigated the association between acute upper respiratory tract infections (URIs) and SSNHL risk. We conducted a case-crossover study by using the longitudinal health insurance database derived from the National Health Insurance Research Database in Taiwan. Individual acute URI between the case and control periods was reviewed. Multivariable conditional logistic regression models were used to estimate the adjusted odds ratios (aORs) of SSNHL risk associated with acute URIs after adjustments for potential confounders. In total, 1131 patients with SSNHL between 2010 and 2013 fulfilled our inclusion criteria and were included. The aOR (95% confidence interval [CI]) for SSNHL was 1.57 (1.20–2.05) in relation to acute URIs one month before the index date. Moreover, the aORs (95% CIs) of the female and young to middle-aged (≤65 years) populations were 1.63 (1.13–2.36) and 1.76 (1.29–2.40), respectively. In addition, the association between SSNHL and acute URIs was decreased over time. The aOR for SSNHL was 1.25 (1.01–1.56) in relation to acute URIs three months before the index date. Acute URIs increase SSNHL risk and are a potential risk factor for SSNHL. The establishment of a feasible health policy for the prevention of acute URIs is crucial for SSNHL prevention, particularly in female, and young to middle-aged populations.

## 1. Introduction

Sudden sensorineural hearing loss (SSNHL) is defined by the National Institute on Deafness and other Communication Disorders as a rapid deterioration (<3 days) of >30 decibels in sensorineural hearing in at least three contiguous frequencies without any identifiable cause [1]. Such hearing loss is mostly unilateral and varies in severity from a mild loss to profound deafness. Its incidence has been estimated to be approximately 5 to 20 per 100,000 persons per year [2,3]. The etiology of SSNHL is still unknown, and some possible pathologic processes, such as viral infections, vascular infraction, cochlear membrane disruption, immune-mediated mechanisms, and abnormal cellular stress responses within the cochlea have been proposed [4].

Several risk factors and underlying medical conditions, such as smoking, cardiovascular disease, chronic otitis media, thyroid diseases, and susceptibility to common colds, have appeared to be positively associated with SSNHL [5,6,7,8]. In addition, one-third of all patients with SSNHL have a history of a previous or concurrent acute upper respiratory tract infection (URI) [9,10] and the number of patients with SSNHL slightly increased from winter to spring [11]. A better understanding of the role of acute URIs in SSNHL etiology may result in new strategies for its prevention because the effective method of preventing acute URIs is handwashing to maintain hygiene [12,13]. Therefore, health policy would be easily established and implemented for the prevention of acute URIs and related comorbidities.

Hearing is crucial for everyone, particularly young people who expect to lead a working life to support their families and society. To understand the avoidable etiology of SSNHL is valuable to establish health policies for its prevention because such hearing loss may become permanent [14]. Therefore, we conducted a large population-based study to explore the causal relation between acute URIs and SSNHL using a case-crossover design.

## 2. Patients and Methods

### 2.1. Data Source

This study was based on the longitudinal health insurance database (LHID 2005) derived from Taiwan’s National Health Insurance Research Database (NHIRD). The LHID 2005 consists of a random sample of 1 million people selected from the 22 million people insured by the National Health Insurance Program in 2005, and the follow-up period was from 1 January 1996 to 31 December 2013. The NHIRD is a nationwide claims database covering 23 million individuals (approximately 99% of the residents in Taiwan) who are beneficiaries of Taiwan’s mandatory National Health Insurance Program. The NHIRD includes comprehensive information on patient demographics, clinical diagnoses, medical expenditures, and prescription records [15]. This study protocol was approved by the Institutional Review Board of Taipei Medical University (TMU-JIRB No. 20151033).

### 2.2. Patients

Patients in the LHID 2005 who received their first diagnosis of SSNHL (International Classification of Diseases, Ninth Revision, Clinical Modification [ICD-9-CM] code 388.2) between 2010 and 2013 were identified as study participants. SSNHL diagnosis was defined as SSNHL as the main diagnosis at discharge or an SSNHL diagnosis (ICD-9-CM code 388.2) confirmed at least twice in outpatient visits. To ensure diagnostic validity, only patients who were diagnosed with SSNHL by otorhinolaryngologists repeatedly during two consecutive outpatient visits were included [16,17]. We excluded patients with a diagnoses of vestibular schwannoma (ICD-9-CM code 388.5, 225.1), Meniere disease (ICD-9-CM code 386.XX), acute or chronic otitis media (ICD-9-CM code 381.XX, 382.XX), malignant disease, and using ototoxic drugs (such as gentamicin, streptomycin, and tobramycin) (ATC code J01GB03, J01GA, J01GB01) during case period. The date of the first diagnosis of SSNHL was defined as the index date.

### 2.3. Study Design

**Figure 1:**
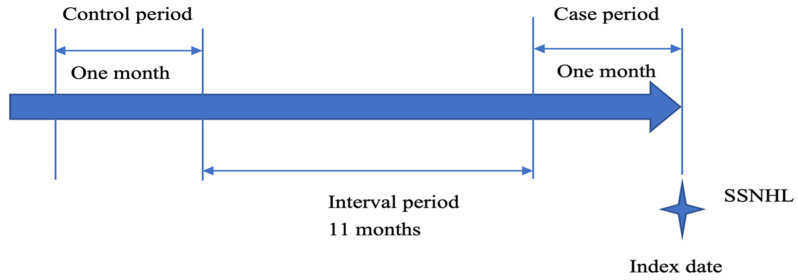
Timeline of case and control periods for the same individuals. (Abbreviations: SSNHL, sudden sensorineural hearing loss).

A case-crossover design was adopted to elucidate the relationship between acute URIs and SSNHL. The case-crossover design is a well-established method to evaluate whether exposure to acute URIs was intermittent, whether the effect of exposure was immediate, and whether the outcome was abrupt [18,19]. In this study, we used a 1-month period as the exposure time window, with the 1st to the 30th day before the index date defined as the case period. To reduce the risk of confounding by seasonal fluctuations, we chose 1 year before the index date as the control period for the same individuals (Figure 1). Because the control period was specific for each patient, time-invariant between-person confounding factors, such as genetic vulnerability or chronic medical conditions, were automatically controlled.

### 2.4. Definition of Acute URI Exposure

Exposure to acute URIs was defined as a diagnosis of acute nasopharyngitis, acute pharyngitis, or acute respiratory tract infection (ICD-9-CM codes 465.9, 465.0, or 460, respectively) [20,21].

### 2.5. Statistical Analysis

Although the case-crossover design automatically controls patients’ pre-existing chronic illness and vulnerability to SSNHL, some time-variant factors, particularly medications, could still confound the results. Therefore, time-variant covariates, including ototoxic drugs such as loop diuretics (e.g., furosemid), anti-inflammatory drugs (nonsteroidal anti-inflammatory drugs), and vascular protection drugs (e.g., statins and anti-platelet drugs), were adjusted in the analysis. A paired *t*-test was used for comparing the control and case period of medication dosage (Table 1).

The standard analysis of case-crossover studies is by conditional logistic regression. Thus, we applied multivariable conditional logistic regression models to estimate the association between acute URIs and SSNHL risk while adjusting important time-variant covariates [adjusted odds ratios (aORs) and 95% confidence intervals (95% CIs)]. Case period was defined as 1st to the 30th day before index date and control period was defined as 366th to 396th day before index date. We can then compare the occurrence of acute URIs in case period versus control period to estimate the risk of acute URIs on SSNHL events. The analyses were stratified by age, gender, chronic medication conditions such as hypertension (ICD-9-CM codes 401.0, 401.1, 401.9), diabetic mellitus (ICD-9-CM codes 250.20, 250.22), dyslipidemia (ICD-9-CM codes 272.2, 272.4, 272.9), end-stage renal disease (ICD-9-CM code 585.6), chronic obstructive pulmonary disease (ICD-9-CM codes 491, 492 or 496), hyperthyroidism/hypothyroidism (ICD-9-CM codes 242.XX/244.XX), and charlson comorbidity index [22,23]. All statistical analyses were performed using Statistical Analysis System (SAS) (version 9.4; SAS Institute, Inc., Cary, NC, USA).

### 2.6. Sensitivity Analyses

Sensitivity analyses were performed to test the influence time of acute URIs on SSNHL. The analyses were repeated by varying the exposure time window to avoid misclassification bias. A longer exposure time window of 2 months (case: control period = 1–60: 366–426 days) and 3 months (case: control period = 1–90: 366–456 days) were explored.

## 3. Results

### 3.1. Study Cohort

**Figure 2:**
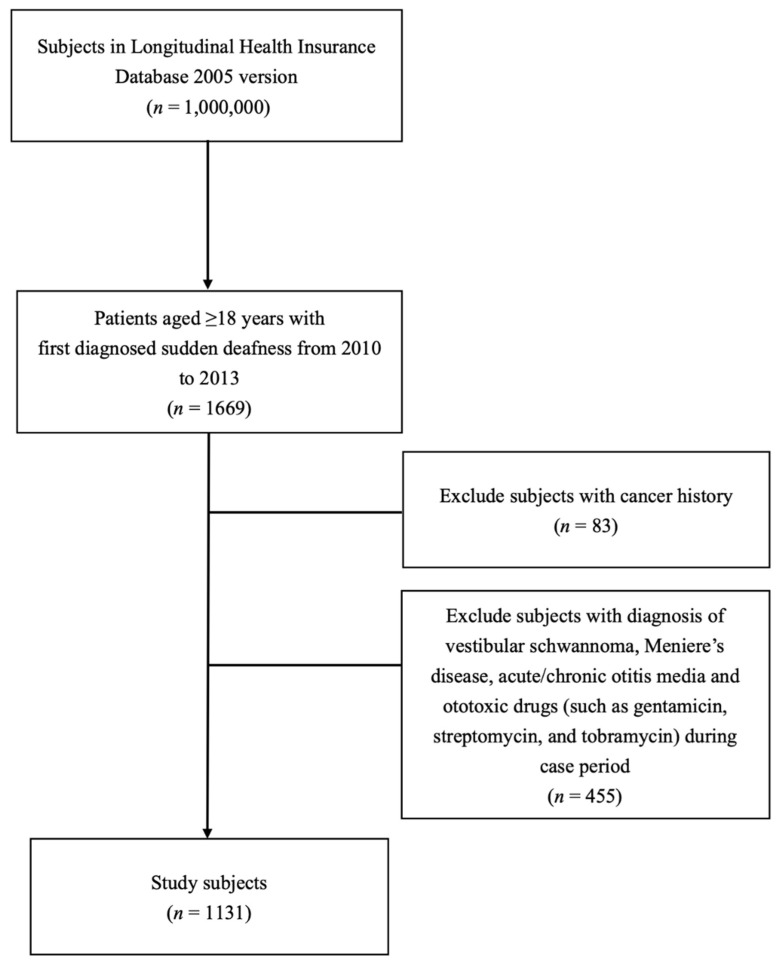
Flowchart of the study.

The flow chart of inclusion and exclusion criteria is presented in Figure 2, and 1131 patients who received a first diagnosis of SSNHL between 2010 and 2013 fulfilled our eligibility criteria. The demographic and clinical characteristics of the study participants are detailed in Table 1. The mean age of patients with SSNHL in this study was 52.8 ± 15.6 years, and 45.5% of them were women.

### 3.2. Conditional Logistic Regression Models of Acute URIs and SSNHL Risk

One hundred and thirty-nine SSNHL patients had suffered from acute URIs one month before they were diagnosed with SSNHL (case period) and no acute URIs episode occurred one year prior to the index date (control period), whereas 90 SSNHL patients suffered from acute URI in the control period but no acute URIs episode in the case period. Comparison of the case and control periods within individual revealed that the acute URIs was significantly associated with increased risk of SSNHL (cOR, 1.54: 95% CI 1.18–2.01). After adjustment for potential confounding medications use, participants with acute URIs showed a 1.57-fold increased risk of SSNHL within 1 month (Table 2).

### 3.3. Sensitivity Analysis of Varying Exposure Time Window

In the sensitivity analysis, the association between acute URIs and SSNHL was weaker, while the exposure time window was longer, including 2 months (aOR, 1.36; 95% CI, 1.09–1.71) and 3 months (aOR, 1.25; 95% CI, 1.01–1.56) before the index date. The hazardous influence of acute URIs on SSNHL decreased over time, but it was still significant (Table 3).

### 3.4. Forest Plots of aORs for SSNHL Stratified by Sex and Age

**Figure 3:**
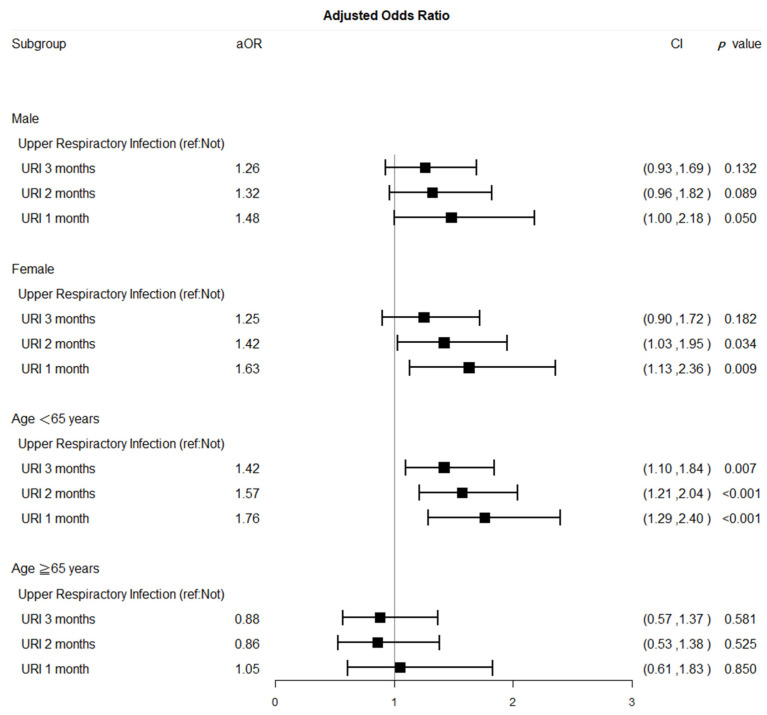
Forest plots of adjusted odds ratios for SSNHL comparing the different exposure time windows for acute URI and control groups by sex and age. (Adjusted covariates of ototoxic drugs, cholesterol-lowing drugs, and anti-platelet drugs listed in Table 1; Abbreviations: SSNHL, sudden sensorineural hearing loss; URI, upper respiratory tract infection; aOR, adjusted odds ratio; CI, confidence interval; ref., reference group; y, years old).

Forest plots of aORs for SSNHL comparing the different exposure time windows for acute URIs and the control groups by sex and age are presented in Figure 3. SSNHL risk was higher in patients aged ≤65 years (aOR, 1.76; 95% CI, 1.29–2.40) and in female patients (aOR, 1.63; 95% CI, 1.13–2.36) within 1 month after an acute URI episode (Figure 3).

## 4. Discussion

This is the first case-crossover design study to elucidate the causal relation between acute URIs and SSNHL. After conditional logistic regression models of acute URIs and SSNHL risk within one month were established, the present study demonstrated that acute URIs indeed increased SSNHL risk by 57% (Table 2 and Table 3). The forest plots of aORs for SSNHL comparing the different exposure time windows for acute URIs with control groups matched for sex and age show that female and young to middle-aged populations were susceptible to acute URI–related SSNHL (Figure 3 and Supplemental Figure S1). The aORs for female and young to middle aged population were higher than those for the male and elderly populations, particularly during the first month after exposure to acute URI. One may wonder why not perform the analysis on three groups (young, middle, and old ages) instead of two groups (young to middle age group and old age group). The definition of age range of young and middle age groups is quite controversial [24,25,26]. In this study, we defined patients who were first diagnosed with SSNHL between 18–39 years old as young age group, between 40–64 as middle age group, and older than 65 years old as old age group. The middle age groups can also be defined by 45–64 years or 50–64 years. We noted that the results of subgroup analysis of acute URIs and SSNHL risk in young, middle, or old age groups were varied according to different definitions of young and middle age groups as shown in Supplemental Figure S1 (young age as 18–39 years), Supplemental Figure S2 (young age as 18–44 years), and Supplemental Figure S3 (young age as 18–49 years). The risk of SSNHL in young age group was significant regardless of age definition; however, the risk of SSNHL in middle age group varied by age definition. The middle age group had a trend of increasing risk, aOR = 1.32 in 40–64 years (*p*-value = 0.088), aOR = 2.71 in 45–64 years (*p*-value ≤ 0.01), aOR = 2.72 in 50–64 years (*p*-value ≤ 0.01) in Supplemental Figures S1–S3. We considered that the trend of increased risk for SSNHL in young to middle age groups altogether was quite robust, though the point estimate could be a bit fluctuated due to smaller sample size in each different defined age group. Thus, in the presentation of current results, we elected to divide our subjects into young to middle age group (18–64 years old) and old age group (≥65 years old) in Figure 3.

Possible reasons and mechanisms are that adult women possess stronger innate and adaptive immune responses than men, but increased susceptibility to inflammatory and autoimmune diseases [27,28]. This explanation is compatible with our finding that the female participants (aOR = 1.63, 95% CI = 1.13–2.36) were more predisposed to SSNHL within one month of acute URI exposure than male participants were (aOR = 1.48, 95% CI = 1.00–2.48; Figure 3). In addition, our study revealed no significant association between acute URIs and SSNHL in elderly patients (age ≥ 65 years; Figure 3). The possible reasons are that the hearing ability of the elderly population (≥65 years) gradually declines, meaning that these patients may be unaware of the symptoms of sudden hearing loss and do not seek care. In addition, SSNHL risk might be attributable to acute URI attack involving acute inflammation accompanied by vasculitis or other episodes of acute inflammation of inner ear structures. With a decrease in the acute inflammation in URIs (in the exposure time window of two or three months), SSNHL risk also decreases. SSNHL is characterized by acute sensorineural hearing loss that is nearly always unilateral and occurs within a 72-h period [29]. Most cases of SSNHL are idiopathic, and the prognosis for hearing recovery depends largely on hearing loss severity [14]. SSNHL mostly occurred between 50 to 60 years of age with no sex preference [30,31]. The characteristics of patients with SSNHL in this study was compatible with previous studies. In our study, the mean age of patients with SSNHL was 52.8 years and revealed slight male preponderance with a male-to-female ratio of 1.2:1. To date, the etiology of sudden SSNHL is unclear, and its presentation represents a diagnostic challenge to primary health care professionals and even to otolaryngologists [14]. Although SSNHL might be associated with various identifiable causes, the majority of cases are idiopathic [32]. Postulated causes of idiopathic SSNHL include viral cochleitis, cochlear membrane disruption, microvascular occlusion, autoimmune disorders [32]. Another rarely mentioned postulated cause of idiopathic SSNHL might be acute URIs contributing to inflammation-related cochleitis, a hypercoagulable state, and autoimmune disorders [32,33,34]. To investigate the association between acute URIs and SSNHL, we conducted a case-crossover study to estimate the risk of acute URIs for SSNHL.

Acute URI is a benign self-limited syndrome representing a group of diseases caused by members of several virus families. It is the most frequent acute illness in the United States and throughout the industrialized world [35]. The term “common cold” refers to a mild upper respiratory viral illness. Acute URI (common cold) is a distinct entity from acute bronchitis, acute bacterial rhinosinusitis, allergic rhinitis, and pertussis [35]. Almost all acute URIs are self-limited but some may result in critical complications, such as acute exacerbation of chronic obstructive pulmonary disease or asthma, pneumonia, and congestive heart failure [36,37]. Moreover, acute URIs may trigger cytokine and chemokine production [38,39] that result in injury to vascular endothelial cells and contribute to several diseases, such as stroke, hypertension, and myocardial infraction [40,41,42].

The potential mechanism by which acute URIs lead to SSNHL is vascular endothelium injury caused by inflammation in acute URIs [40,41,43]. Vascular endothelial cells play a crucial role in circulation, and they control vascular tone (blood flow), leukocyte activation, platelet aggregation, and adhesion [40,44]. When vascular endothelial cells are injured in acute URIs, the synthesis and bioactivity of vasodilators (such as nitric oxide and prostacyclin) are reduced and the production of several proinflammatory cytokines (such as interleukin-1, interleukin-6, and tumor necrosis factor-α) is increased due to alteration in the coagulation and fibrinolytic systems [40,41,43,45]. Respiratory viruses such as rhinovirus, influenza A, adenoviruses, and coronaviruses may directly or indirectly damage the human vascular endothelium and induce thrombotic complications [46,47,48]. These immune responses and inflammatory processes after respiratory tract infections may be the main mechanism of SSNHL. Furthermore, some chronic inflammatory diseases such as diabetic mellitus and dyslipidemia were also important factors in the pathogenesis of SSNHL [49,50] and some studies disclosed SSNHL patients increased cardiovascular risk such as stroke and acute myocardial infraction [16,51,52]. Hence, the SSNHL may share a similar pathogenesis of microangiopathy with cardiovascular disease and this vascular theory of SSNHL was also consistent with our inference from this study. On the other hand, the acute inflammatory disease like acute URIs induced SSNHL had greater impact on the female population, whereas chronic inflammatory disease like diabetic mellitus and dyslipidemia that may influence more the male population, who without estrogen-related vascular protective effect, result in cochlear microvascular compromises. This hypothesis of acute and chronic inflammatory disease-induced vascular endothelium injury may explain why SSNHL had equal sex distribution, but needs further study to prove it.

Hearing loss is a leading cause of disability worldwide [53]. According to the Institute for Health Metrics and Evaluation’s Global Burden of Disease Study in 2019, hearing loss accounts for 1.58% of total disability-adjusted life years, with an annual increase of 0.81% [54]. SSNHL is nonpreventable because its etiology is unclear and its treatments are diverse. SSNHL treatments include steroids, vasodilators, antiviral agents, diuretics, antioxidants, and hyperbaric oxygen [4]. Treatment modalities for SSNHL are aimed at inhibiting the inflammatory process and increasing blood flow and oxygen delivery to the inner ear [4]. Of these treatments, systemic steroid therapy is recognized as the primary and most effective because it inhibits inflammation related to cochleitis, a hypercoagulable state, and autoimmune disorders [55,56]. However, approximately 30 to 50% of patients have shown poor responses to systemic steroid therapy, and approximately one-third of patients do not recover completely [57,58]. Therefore, the incidences of SSNHL must be reduced and awareness of SSNHL must be increased by elucidating the risk factors for SSNHL. The majority of URIs are transmitted by hand contact [12]; cold-inducing viruses may remain viable on human skin for up to two hours [12,13]. Acute URIs might be a risk factor for SSNHL, according to our findings (Table 2 and Table 3); proper use of mask and adequate handwashing can be assumed help prevent the spread of URIs and decrease SSNHL risk [12,13]. Hand hygiene should be emphasized in public health policy for the prevention of avoidable hearing loss [12,13].

In summary, the pathogenesis of SSNHL is still controversial and SSNHL is more like a syndrome rather than a disease because of a range of different causes. From our study, we may infer that acute URIs had its role in development of SSNHL, particularly in female and young to middle-aged (<65 years) populations. Future public health policies for the prevention of acute URIs related to SSNHL will pay more attention to the vulnerable groups of women and young to middle-aged people (Supplemental Table S1, Figure 3, and Supplemental Figures S1–S3). Hearing is important for everyone, particularly young people who expect to lead a long-term working life to support their families and society. Therefore, women and young to middle-aged people should take notice of the symptoms like aural fullness, tinnitus, or sudden hearing loss after acute URIs and receive comprehensive examination and treatment as soon as possible.

The strengths of our study are its evaluation of the risks of acute URIs and SSNHL; previous studies have not had a sufficiently large sample size. Moreover, our study is the first to reveal that acute URI is a significant risk factor for SSNHL, particularly in female, and young to middle aged populations. Moreover, we also performed sensitivity analysis (Table 3) of various exposure time windows for acute URIs and SSNHL risk to show that acute URIs related to acute inflammation contribute SSNHL risk, which fades with time, to confirm the acute effects of URIs instead of its chronic effects on SSNHL risk. Our findings could be valuable for establishing future health policies for SSNHL prevention. In particular, female and young to middle-aged populations must pay attention to the proper use of mask and hand hygiene for SSNHL prevention. In addition, the acute effects of URI within one month is crucial for the therapeutic time window. The increase in SSNHL risk within one month of exposure to acute URI means that early treatments [4] of steroids, vasodilators, antiviral agents, diuretics, antioxidants, or hyperbaric oxygen for acute URI–related inflammation might improve the outcome of SSNHL.

This observational study has some limitations. First, SSNHL diagnosis was based on information in the NHIRD; although previous studies [59,60,61] have validated that diseases in the NHIRD are accurate, the validity of SSNHL diagnosis in the database has not been proven. Even though the diagnoses of SSNHL in our study were all made by professional otolaryngologists in Taiwan, the misclassification bias (accuracy of diagnoses of SSNHL and acute URIs) is the biggest limitation of our study. Therefore, we excluded participants with a concurrent diagnosis of diseases with hearing loss symptoms, such as acute/chronic otitis media, Meniere disease, and vestibular schwannoma, to ameliorate the validity of SSNHL diagnosis. In addition, the acute rhinitis, pharyngitis, sinusitis, tonsillitis, epiglottitis, laryngitis, influenza infection are all manifestations of acute URIs. In this study, we narrowed down the definition of the acute URI as acute nasopharyngitis, acute pharyngitis, or acute respiratory tract infection. Consequentially, the result of this study may not be overestimated. Moreover, the NHIRD in Taiwan have generally reported positive predictive values of over 70% for various diagnoses [62], despite that the validation study of diagnostic accuracy of acute URIs in NHIRD is lacking. Several studies had used ICD-9 CM code 460, 465.0, 465.9 as acute respiratory tract infection, acute pharyngitis, and acute nasopharyngitis to apply in their research [20,21], with robust findings, it seems reasonable to use the ICD-9 CM code 460, 465.0, 465.9 to present acute URIs episode in this study. Second, although we adopted a case-crossover study design to remove time-invariant confounders and control time-variant medications, some potential confounding factors such as life style and personal habit changes might have influenced our study results. Smoking and heavy drinking were both known as risk factors of SSNHL [7]. Third, capturing all exposures to acute URIs among the study participants was impossible. Not all individuals with acute URIs seek medical help, and the association between acute URIs and SSNHL risk may be underestimated. Nevertheless, the underdiagnosis of acute URIs in recruited participants only led to the underestimation of SSNHL risk and did not affect the hypothesis. Thus, our conclusions could not be overturned in the study. Finally, our study enrolled only Asian people, and whether the results can be extrapolated to other countries or non-Asian populations requires further investigation. However, no evidence showed differences in ethnicity for acute URIs or SSNHL.

## 5. Conclusions

This study revealed that acute URIs increase SSNHL risk, with the highest risk evident within the first month after exposure to an acute URI. Female and young to middle-aged (<65 years) populations are susceptible to SSNHL after an acute URI episode in Taiwan.

## Figures and Tables

**Table 1:** Characteristics of Case-Crossover Patients with SSNHL Having and Not Having Acute URI Exposure (1-Month Period as the Exposure Time Window).

Variables	Case Period	Control Period	*p* Value *
Age at first SSNHL diagnosis (years), N (%)			
18–39	261 (23.1%)		
40–64	622 (55.0%)		
≥65	248 (21.9%)		
Sex			
Female	515 (45.5%)		
Male	616 (54.5%)		
Disease			
Hypertension	396 (35.0%)		
Diabetes mellitus	219 (19.4%)		
Dyslipidemia	323 (28.6%)		
End-stage renal disease	12 (1.1%)		
Chronic obstructive pulmonary disease	104 (9.2%)		
Hyperthyroidism/hypothyroidism	65 (5.7%)		
Charlson Comorbidity Index			
0	1043 (92.2%)		
1–2	69 (6.1%)		
≥3	19 (1.68%)		
Medication dosage, DDD (mean ± SD)			
Statins	2.0 ± 7.9	1.8 ± 7.8	0.167
Antiplatelet	(3.6 ± 12.1)	(3.2 ± 11.5)	0.424
NSAIDs	(3.1 ± 8.0)	(2.7 ± 8.2)	0.506
Furosemide	(0.9 ± 10.1)	(0.7 ± 3.8)	0.233

Abbreviations: DDD, defined daily dose; SSNHL, sudden sensorineural hearing loss; SD, standard deviation; N, number; NSAID, nonsteroidal anti-inflammatory drug. * Analyzed using paired t test, significance at *p* < 0.05.

**Table 2:** Conditional Logistic Regression Models of Acute URIs and Risk of SSNHL within 1 Month.

	Case Period (*n*) ^a^	Control Period (*n*) ^b^	cOR (95% CI)	aOR (95% CI) ^c^
Acute URIs	139	90	1.54 (1.18–2.01)	1.57 (1.20–2.05)

Abbreviations: aOR, adjusted odds ratio; CI, confidence interval; cOR, crude odds ratio; URI, upper respiratory tract infections; ^a^ Case period: acute URI in the case period but not in the control period; ^b^ Control period: acute URI in the control period but not in the case period ^c^ Adjusted covariates used of ototoxicity drugs, cholesterol-lowing drugs, and anti-platelet drugs listed in Table 1.

**Table 3:** Sensitivity Analysis of Various Exposure Time Windows for Acute URIs and SSNHL Risk.

Exposure Time Window	Case Period 1–30 Days Control Period 365–395 Days		Case Period 1–60 Days Control Period 365–425 Days		Case Period 1–90 Days Control Period 365–455 Days	
	cOR (95%CI) aOR (95%CI) ^a^		cOR (95%CI) aOR (95%CI) ^a^		cOR (95%CI) aOR (95%CI) ^a^	
Acute URIs	1.54 (1.18–2.01)	1.57 (1.20–2.05)	1.36 (1.08–1.70)	1.36 (1.09–1.71)	1.26 (1.01–1.56)	1.25 (1.01–1.56)

Abbreviations: cOR, crude odds ratio; aOR, adjusted odds ratio; URI, upper respiratory tract infections; CI, confidence interval; ^a^ Adjusted covariates used of ototoxic drugs, cholesterol-lowing drugs, and anti-platelet drugs listed in Table 1.

## Data Availability

Restrictions apply to the availability of these data. Data were obtained from the Health and Welfare Data Science Center and are available with the permission of the Health and Welfare Data Science Center, Taiwan.

## Supplementary Materials

The following are available online at https://www.mdpi.com/1660-4601/18/20/10745/s1, Figure S1: Forest plots of adjusted odds ratios for SSNHL comparing the different exposure time windows for acute URI and control groups by sex and age (18–39, 40–64, and ≥65 years old), Figure S2: Forest plots of adjusted odds ratios for SSNHL comparing the different exposure time windows for acute URI and control groups by sex and age (18–44, 45–64, and ≥65 years old), Figure S3: Forest plots of adjusted odds ratios for SSNHL comparing the different exposure time windows for acute URI and control groups by sex and age (18–49, 50–64, and ≥65 years old), Table S1: Subgroup analysis of different age groups for acute URIs and SSNHL risk in different exposure windows.

## Abbreviations

aOR	adjusted odds ratio
CI	confidence interval
cOR	crude odds ratio
SSNHL	sudden sensorineural hearing loss
URIs	upper respiratory tract infections
DDD	defined daily dose
SD	standard deviation
LHID	longitudinal health insurance database
NHIRD	National Health Insurance Research Database
ICD-9-CM	International Classification of Diseases, Ninth Revision, Clinical Modification
