# Supplementary material for: Association of Acute Upper Respiratory Tract Infections with Sudden Sensorineural Hearing Loss: A Case-Crossover, Nationwide, Population-Based Cohort Study
Source: Int J Environ Res Public Health. 2021 Oct 13;18(20):10745. doi: 10.3390/ijerph182010745 (PMC8535477; doi:10.3390/ijerph182010745)
Supplement: Supplementary file 1 [file ijerph-18-10745-s001.zip › ijerph-1358437-supplementary.pdf]

Supplemental Figure S1. Forest plots of adjusted odds ratios for SSNHL comparing the different exposure time windows for acute URI and control groups by sex and age (18-39, 40-64, and ≥65 years old)

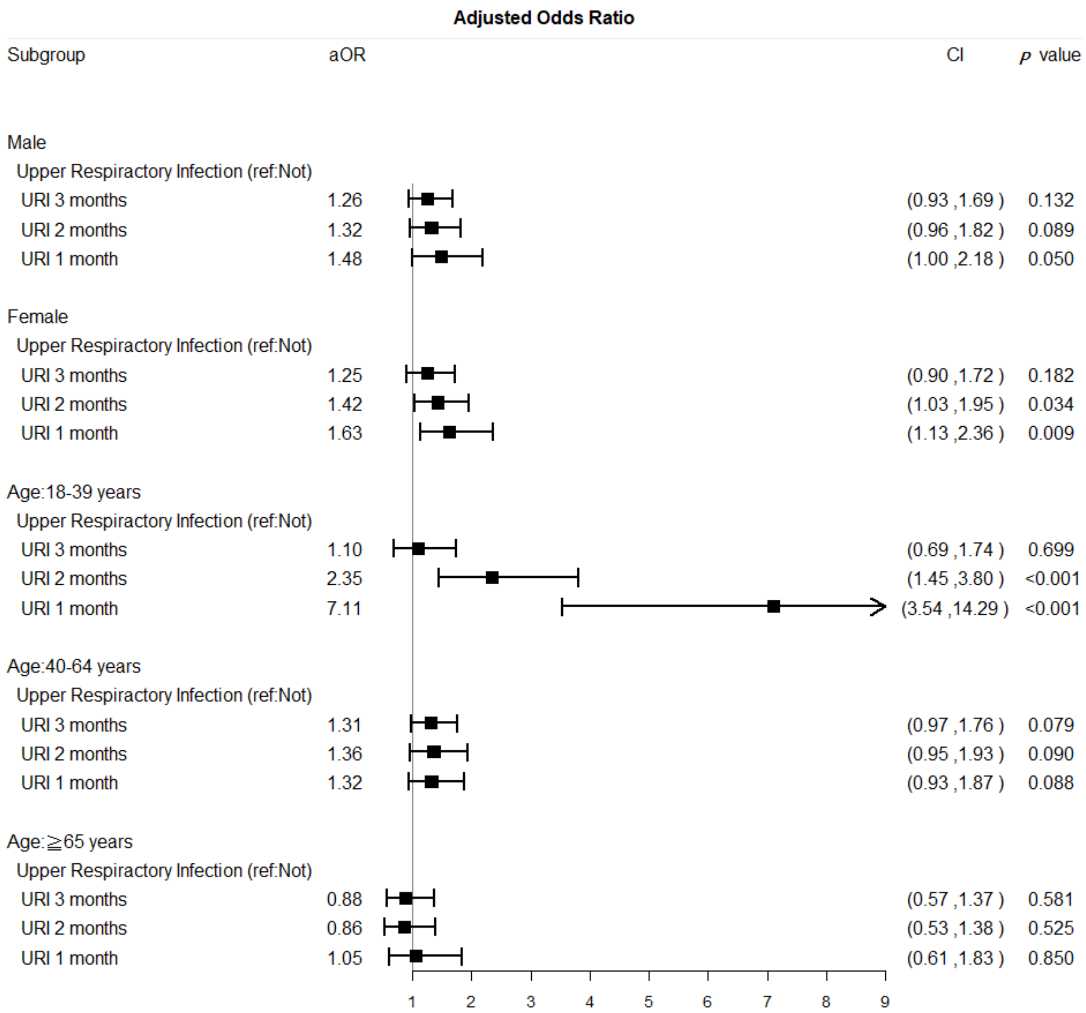


Supplemental Figure S2.

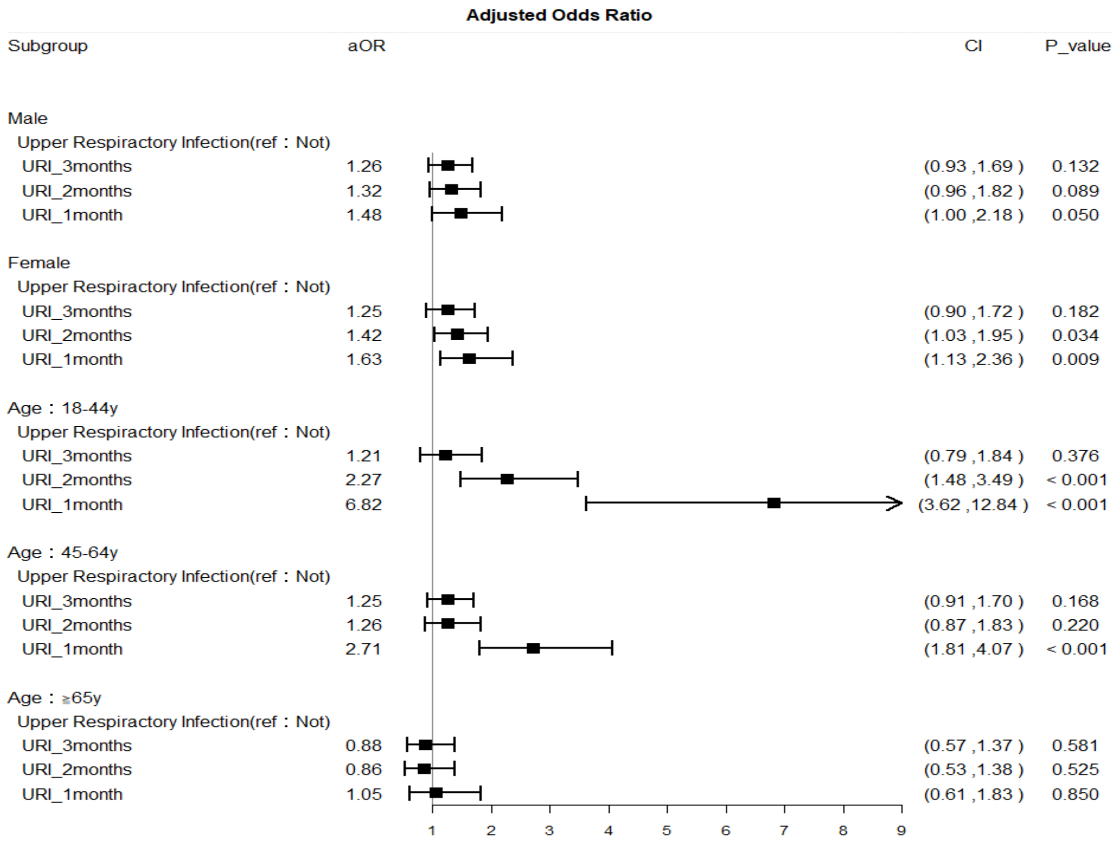


Supplemental Figure S3.

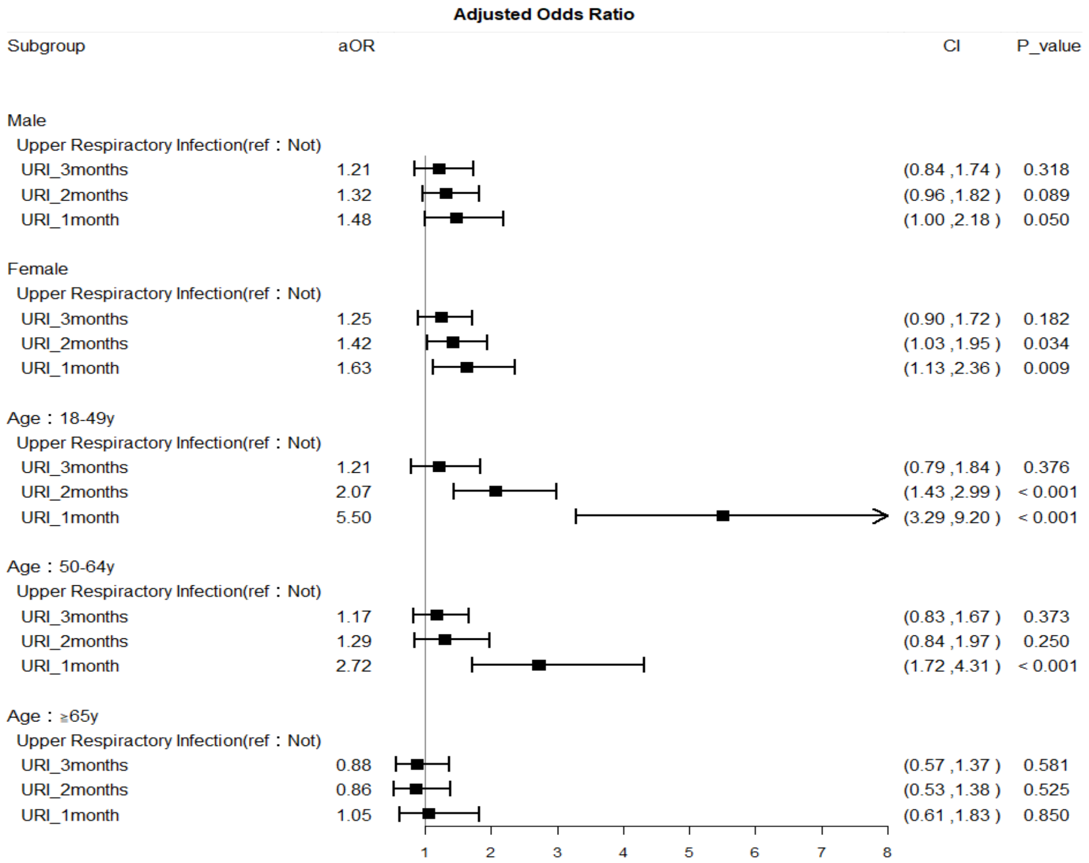

Supplemental Table 1. Subgroup analysis of different age groups for acute URIs and SSNHL risk in different exposure windows

| | Exposure window | One month | *P* value* | Two months | *P* value* | Three months | *P* value* |
|---|---|---|---|---|---|---|---|
| Age | | aOR(95%CI) [a] | | aOR(95%CI) [a] | | aOR(95%CI) [a] | |
| 18-39 y/o | | 2.03(1.01-4.05) | 0.045 | 1.61(0.97-2.67) | 0.063 | 0.99(0.53-1.84) | 0.965 |
| 40-64 y/o | | 1.07(0.61-1.88) | 0.809 | 1.19(0.79-1.80) | 0.397 | 1.16(0.67-2.01) | 0.595 |
| ≥ 65 y/o[+] | | 1 | | 1 | | 1 | |

Abbreviations: aOR, adjusted odds ratio; CI, confidence interval; SSNHL, sudden sensorineural hearing loss; URI, upper respiratory tract infections

[+]≥ 65 y/o as reference group

*Analyzed using logistic regression model with Wald chi-square statistics test, significance at $P< 0.05$

[a] Adjusted covariates used of ototoxicity drugs, cholesterol-lowing drugs, and anti-platelet drugs listed in Table 1.
